# CD56^dim^CD16^high^ and CD56^bright^CD16^−^ cell percentages associated with maximum knee extensor strength and incidence of death in elderly

**DOI:** 10.1186/s40064-016-1884-3

**Published:** 2016-03-01

**Authors:** Hidenobu Senpuku, Hideo Miyazaki, Akihiro Yoshihara, Saori Yoneda, Naoki Narisawa, Taketo Kawarai, Naoki Nakagawa, Motohiko Miyachi, Akio Tada, Goichiro Yoshida, Mieko Shimada, Masaharu Ohashi, Mamoru Nishimuta, Yasuo Kimura, Yutaka Yoshitake

**Affiliations:** Department of Bacteriology, National Institute of Infectious Diseases, 1-23-1 Toyama, Shinjuku-ku, Tokyo 162-8640 Japan; Department of Oral Health Science, Graduate School of Medical and Dental Science, Niigata University, Niigata, Niigata 950-2181 Japan; Laboratory of Physical Education, Sports Management Research Center, School of Information-oriented Management, SANNO University, Setagarya-ku, Tokyo 158-8630 Japan; Department of Health Promotion and Exercise, National Institute of Health and Nutrition, Shinjuku-ku, Tokyo 162-8640 Japan; Department of Health Science, Hyogo University, Kakogawa, Hyogo 675-0101 Japan; National Institute of Fitness and Sports in Kanoya, Kanoya, Kagoshima 891-2311 Japan; Laboratory of Physical Education, Chiba College of Health Science, Chiba, Chiba 261-0014 Japan; Faculty of Education and Human Science Physical Education, Health and Sport Science, Institute of Humanities, Social Science and Education, Niigata University, Niigata, Niigata 950-2181 Japan; Faculty of Culture and Education, Saga University, Saga, Saga 840-8502 Japan

**Keywords:** Elderly, Physical fitness, Maximum bilateral knee extensor strength, NK cell, CD56^dim^CD16^high^ cell, CD56^bright^CD16^−^ cell

## Abstract

Physical fitness is an indicator of systemic well-being in humans. Little is known about the role of physical fitness for maintaining systemic health in the elderly. Here, we study elderly subjects to determine the relationships between physical fitness and CD56 and CD16 surface NK cell markers on peripheral blood lymphocytes, as well as to analyze the relationship between the surface markers and incidence of death. We selected 253 independent elderly subjects (122 female; 131 male) who were 79–80 years old. Subjects having a higher proportion of CD56^dim^CD16^high^ within CD56^+^CD16^+^ cells, or ration of CD56^dim^CD16^high^ and CD56^dim^CD16^−^ cells had a significant positive correlation with maximum bilateral knee extensor strength/weight (kg) (r = 0.425; *P* < 0.0001 or r = 0.323; *P* < 0.0001). In contrast, an increased proportion of CD56^bright^CD16^−^ cells within lymphocyte significantly negatively correlated with the maximum bilateral knee extensor strength/weight (kg) (r = −0.290; *P* = 0.0004); and these subjects had a significantly lower mortality during the 5 years following measurement of death. Therefore, we found that a synergistic effect of the right and left leg muscle strength was associated with proportion of matured NK and NKT cells and induced a low proportion of CD56^bright^CD16^−^ cells within lymphocyte. Moreover, the low proportion of CD56^bright^CD16^−^ cells was associated with incidence of death. In conclusion, measurements of physical fitness, the proportion of CD56^dim^CD16^high^ within CD56^+^CD16^+^ cells, the ratio of CD56^dim^CD56^high^ and CD56^dim^CD16^−^ cells, and the proportion of CD56^bright^C16^−^ cells in lymphocytes are important indicators to check elderly health.

## Background

In developed countries, the elderly population is increasing in concurrence with a low birth rate. This has led to an increase in the bed-ridden elderly who have few opportunities to perform daily physical activity. This decreased daily physical activity increases the risk of systemic diseases, e.g., aspiration pneumonia, cardiovascular diseases, hypertension, osteoporosis, type II diabetes and obesity (Chodzko-Zajko et al. 2009; Kohl 2001; Yoneyama et al. 2002). The decline in muscular strength may lead to a reduction in immune function and thus to the loss of resistance to infectious disease, e.g., aspiration pneumonia (Takata et al. 2007; Eickmeyer et al. 2012) and cancer development (Brown et al. 2012; Eickmeyer et al. 2012; Dimeo et al. 2004). Low levels of physical activity are postulated to be a substantial contributor to cancer-related fatigue (Eickmeyer et al. 2012); and interventions to increase physical activity in cancer patients are shown to reduce the severity of this symptom. Physical activity is found to play a role in the prevention of certain malignancies, including breast, colon, and other cancers (Brown et al. 2012).

In athletes, the peripheral blood natural killer (NK, CD16^+^/56^+^) cell numbers are generally normal, although NK cell cytotoxic activity may be higher at rest in athletes compared with non-athletes (Kusaka et al. 1992; Nieman et al. 1995). Others report enhanced in vivo NK cell cytotoxicity after exercise intervention in smaller studies (Nieman et al. 1995; Nieman 2000). Results from a 10-week study among 25 elderly nuns (mean age, 77 years old) where the intervention consisted of 20–50 min of brisk walking 3 days per week suggested improved NK cell cytotoxicity but no effect on in vitro T-lymphocyte proliferation (Fahlman et al. 2000). Additionally, it has been largely suggested that moderate exercise training has no effect on T-lymphocyte proliferation, as well as the numbers and percentage of NK cells, leukocytes, and lymphocytes (Nieman 2000; Scanga et al. 1998). In contrast, moderate-intense exercise is associated with conditions linked to immunity, including resistance to upper respiratory tract infection (Chubak et al. 2006). There is no consistent correlation observed in the various reports involving exercise and immunity (Moreira et al. 2009).

Physical fitness refers to a physiologic state of well-being allowing individuals to meet the demands of daily living or provides the basis for sport performance, or both. Physical fitness appears to be similar to physical activity in its relationship to morbidity and mortality (Blair and Brodney 1999; Erikssen 2001) but it is stronger in predicting health outcomes than increased physical activity (Blair et al. 2001; Myers et al. 2004; Williams 2001). The assessment of physical fitness is often not feasible or practical in a large population-based investigation. Therefore, additional studies as addressed here are required to determine if there is a correlation between physical fitness and immune cells in a large population.

When a microbe invades, cell-mediated immunity is the first defense mechanism for protection against invaders. With cell-mediated immunity, NK cells respond to cues from sentinel immune cells, including macrophages and pathogen-infected tissues (Biron et al. 1999); and play an important role in the defense against viral infections as well as in tumor surveillance (Lanier 2008). NK cells are also associated with the adaptive immune response through the production of cytokines (Lanier 2008). In humans, NK cells are usually defined as CD3^−^CD56^+^ cells (Lanier et al. 1986), and can be further subdivided based on CD56 expression. CD56^dim^ NK cells constitute the majority (90 %) of peripheral blood NK cells (Cooper et al. 2001a, b) and express high levels of the low-affinity Fc receptor, CD16, and high levels of perforin (Cooper et al. 2001a, b; Ferlazzo et al. 2004). CD56^dim^ NK cells are terminally differentiated cells. These cells express the CD16 marker and are involved in antibody-dependent cellular cytotoxicity. CD16 is associated with the activation of motif-containing adaptor proteins (Lanier 2008). Engagement of CD16 is sufficient to induce IFNγ and TNFα secretion in addition to chemokine secretion. NK cell maturation is characterized by the down-regulation of CD56 and the acquisition of CD16. Reports show NK cell activation is highest when CD56^dim^CD16^+^ activated cells are observed (Dulphy et al. 2008). Therefore, CD56^dim^CD16^+^ cells include the most mature cell type of NK cells (André et al. 2000; Nagler et al. 1989).

NKT (natural killer-like T) cells were originally described as a unique population of T cells with the co-expression of NK cell markers (Golden-Mason et al. 2007; Mercer et al. 2005; Tarazona et al. 2000). A consistent subset of T cells expresses bright CD56; however, CD16 is rarely expressed on T cells in healthy subjects (Lambert et al. 2006). CD3^+^CD16^+^CD56^+^ T cells are not classical invariant NKT cells but are a broader group of T cells matching the original definition of NKT cells (Golden-Mason et al. 2007; Mercer et al. 2005) and have cytotoxic activity (Forner et al. 1994).

Understanding NK and NKT effector functions may lead to the development of improved strategies for the treatment of many diseases. NK and NKT cells are innate immune cells that act as the first line of defense against infection and tumorigenesis. NK cells are essential for tumor rejection (Park et al. 2003; Herberman et al. 1975; Kiessling et al. 1975); and Subleski et al. report that NK cells are an important component for tumor regression in a murine liver tumor model (Subleski et al. 2006). Recent studies support that NK cells play a critical role in tumor control and eradication (Waldhouer and Steinle 2008). An 11-year follow-up study of 3625 of Japanese population was performed for cancer immune-surveillance for NK cells; low NK cell cytotoxicity correlated with an increased risk for cancer (Imai et al. 2000). Thus, NK cell-mediated anti-tumor immunity is required to overcome the development of cancer.

In this study, we found three characteristic cell types, CD56^dim^CD16^high^, CD56^dim^CD16^−^ and CD56^bright^CD16^−^ cells, within the overall lymphocyte population in peripheral blood cells. We performed a correlation analysis between these three cell types, and indicators of physical fitness with incidence of death during 5 years following the initial testing. To clear relationship between the maturation of CD56^+^CD16^+^ cells and physical fitness, the ratios of CD56^dim^CD16^high^ and CD56^dim^CD16^−^ cells were analyzed. We found that the proportion of CD56^dim^CD16^high^ within CD56^+^CD16^+^ cells and the ratio of CD56^dim^CD16^high^ and CD56^dim^CD16^−^ cells were positive immune indicators when compared to physical fitness such as the maximum bilateral extensor strength; proportion of CD56^bright^CD16^−^ cells within lymphocytes showed as a poor outcome indicator leading to increased incidence of death in the very elderly.

## Methods

### Elderly subjects

The elderly population for this study was drawn from the Niigata Study. Briefly, the Niigata Study is a prospective community-based study initiated in 1998 to evaluate the relationships between an individual’s diseases. Questionnaires were initially sent to all inhabitants (n = 4542) age 70 years based on a registry of residents in Niigata City, Japan; all recipients were informed of the purpose of this survey. Among the elderly subjects participating in the Niigata study (n = 600), 367 individuals were 79 years in 2007 and 80 years in 2008 and were involved in the study. We randomly selected 253 independent elderly subjects (122 female; 131 male) age 79–80 years old; this group was limited because our assay to measure the characteristics of CD56^+^CD16^+^ cells in a FACS analysis had a maximum capacity of 40 samples per experimental day. As controls, young people (n = 35) age 18–22 years old were randomly selected; and were students attending Kanoya University and Chiba College of Health Sciences. When more than 40 subjects presented on a sampling day, the first 40 subject’s blood was extracted and their NK cells observed. The participants agreed to have medical and dental examinations, and physical fitness tests; and signed an informed consent form showing the protocol was approved by the Ethics Committees of Niigata University Graduate School of Medical Dental Science, National Institute of Infectious Diseases, National Institute of Health Science, and Chiba College of Health Science. Follow-up surveys were performed every year during June using the same methods as in the initial survey. Body height and weight were recorded and the body mass index (BMI) was calculated in all elderly subjects. All subjects were in good general health and did not require special care. Data for males were similar to data for females and, therefore, mixed data from males and females were analyzed to determine the correlations in physical fitness, immunological parameters and biochemical parameters.

### Physical fitness parameters

We conducted a medical examination followed by six physical fitness tests: maximum hand grip strength (Rantanen et al. 1998; Sonn et al. 1995), maximum unilateral and bilateral knee extensor strength (Rantanen et al. 1999; Sonn et al. 1995), maximum stepping rate for 10 s (Shindo et al. 1987), one-leg standing time with eyes open (Michikawa et al. 2009; Haga et al. 1986; Stones and Kozma 1987), and a 10-m maximum walking speed test (Shinkai et al. 2000). The maximum hand grip strength in both the dominant and non-dominant hand was measured using a Smedley hand dynamometer (DM-100s, Yagami, Inc., Nagoya, Japan). The score used was the best of the trials between both grip strengths. The equipment for the isometric leg extension and hand grip strength tests were determined and calibrated before beginning the test. The maximum unilateral and bilateral isometric knee extensor strength was determined using a portable chair incorporating a dynamometer connected to a load cell. The subject sat on a seat in a vertical position that was adjusted so that he or she sat comfortably with legs hanging vertically and the knees bent at 90°. The measurement of the maximum unilateral knee extension strength was performed twice on the right and left legs alternately, and thereafter the measurement of the maximum bilateral knee extension strength was performed once. The rest interval between contractions of the leg muscles was more than 2 min. The maximum step rate for 10 s was used as an index of agility using an industrial step rate counter (Stepping Counter, Yagami Inc). The subject was instructed to step as fast as possible for 10 s with each leg alternately while in a sitting position. The stepping rate for the left and right leg was summed for this analysis. The one-leg standing time with eyes open was measured. The static balance function was measured with the subject’s eyes open and arms out, standing on one foot with the other off the floor. The score was either the number of seconds when the non-preferred foot was raised and balance was lost (when the subject began to hop around or when the raised foot was lowered to the floor) or when 2 min had elapsed. The subjects performed one trial on each foot and the best score was recorded. The maximum walking speed was measured having the subjects walk at their fastest pace over a 10-m course. The fastest walking speed was used.

### Lymphocyte separation and CD56 and CD16 cell staining

Whole blood (5–7 ml) was collected from subjects in sodium heparin tubes (10 ml VENOJECT; Terumo, Japan) between 10:00 A.M. and 1:00 P.M., before lunch and after resting from recent physical activity. The blood was sent immediately to the National Institute of Infectious Diseases, diluted with an equal volume of Hanks’ Balanced Salt Solution (HBSS) (Gibco Laboratories, Life Technologies; Paisley, UK); then, layered on a Ficoll-Conray density gradient separation solution (Lymphosepar I, Immuno-Biological Laboratories, Gunma, Japan); and centrifuged at 570×*g* for 30 min at room temperature. The peripheral blood mononuclear cell (PBMC) layer was removed and washed twice in HBSS. The PBMCs were stained for markers using PC5-conjugated CD56 and fluorescence isothiocyanate (FITC)-conjugated CD16 mAbs to observe natural NK cells. FITC-conjugated anti-human CD16 (clone 3G8) monoclonal antibodies (mAbs) were purchased from BD PharMingen (San Diego, CA). Phycoerythrin-cyanine 5 (PC5)-conjugated anti-human CD56 and FITC and PC5-conjugated mouse immunoglobulin G1 (IgG1) (clone 679.1Mc7) were purchased from Immunotech (Marseille, France). Separating NK and NKT cells was not performed to reduce the numbers of antibodies for FACS analysis (CD3 expression was not evaluated). For each subject, PC5- and FITC-conjugated IgG1 isotype controls were used. The PBMCs (1 × 10^5^) aliquots were placed in v-bottomed 96-well plates (Coster, Cambridge, MA); incubated with 20 μl mAb in 1 % bovine serum albumin for 60 min at 4 °C; washed three times with 150 μl of HBSS; fixed with 150 μl of 5 % formalin/PBS solution; and stored at 4 °C until flow cytometry analysis was performed. The percentage of FITC- and PC5-positive cells was measured using a FACSCalibur flow cytometer (Becton–Dickinson; Franklin Lakes, NJ); and the data were analyzed using CellQuest Pro software (Becton–Dickinson). Lymphocytes were defined and separately gated on the basis of forward light scatter (FSC) and side light scatter (SSC) for further analysis. Furthermore, the proportions of the major subsets of cells stained by antibodies were determined using quadrant analysis and gating of individual area on the basis of the first-gated lymphocytes.

**Fig. 1:**
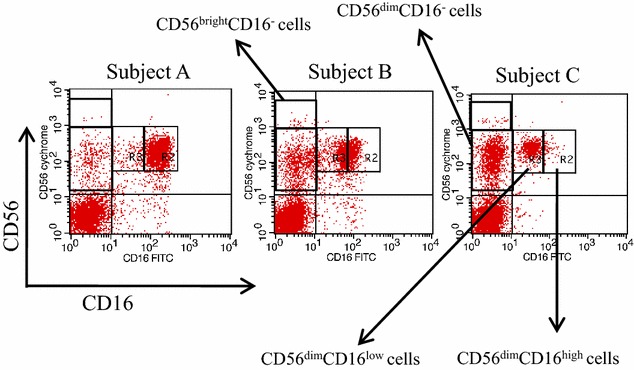
Analysis of mature and less-mature NK cells in elderly subjects. Lymphocytes stained using anti-CD56 and anti-CD16 antibodies were gated and analyzed using flow cytometry. The *left circle* indicates the CD56^dim^CD16^high^ cells, which are less mature NK cells. The *right circle* indicates the CD56^dim^CD16^low^ cells, which are mature NK cells

Two types of cell groups (CD56^dim^CD16^high^ cells and CD56^dim^CD16^low^ cells) were found inside two individual squares in upper and right square using quadrant analysis (cells at either higher or lower than the median fluorescence intensity and the same intensity level as the CD16-positive population on the CD56^dim^ cells) (Fig. 1a–c). Further, the two types of cell groups (CD56^dim^CD16^−^ cells and CD56^bright^CD16^−^ cells) were also found inside two individual squares in upper and left square (Fig. 1a–c). The dots in the left square show the numbers of CD56^dim^CD16^−^ cells and CD56^bright^CD16^−^ cells, which may be the less mature NK and NKT subpopulations; the dots in the right squares show the numbers of CD56^dim^CD16^bright^ cells, which may be the mature NK and NKT subpopulations in each subject (A–C). Variable levels of CD16 cells with CD56^dim^ or CD56^high^ expressions were found in the elderly subjects. The proportions of CD56^+^CD16^+^ cells, CD56^dim^CD16^high^ cells, CD56^dim^CD16^low^ cells, CD56^dim^CD16^−^ cells or CD56^bright^CD16^−^ cells within the gated lymphocytes in guardant analysis (CD56^+^CD16^+^/lymphocytes, CD56^dim^CD16^high^/lymphocytes, CD56^dim^CD16^low^/lymphocytes, CD56^dim^CD16^−^/lymphocytes or CD56^bright^CD16^−^/lymphocytes) were analyzed for each subjects using CellQuest Pro software. The proportions of CD56^dim^CD16^high^ cells or CD56^dim^CD16^low^ cells within the gated CD56^+^CD16^+^ cells in guardant analysis (CD56^dim^CD16^high^/CD56^+^CD16^+^ or CD56^dim^CD16^low^/CD56^+^CD16^+^) were calculated for each subject by division between proportions of CD56^dim^CD16^high^/lymphocytes or CD56^dim^CD16^low^/lymphocytes and CD56^+^CD16^+^/lymphocytes. The ratio of CD56^dim^CD16^high^ cells and CD56^dim^CD16^−^ cells (CD56^dim^CD16^high^/CD56^dim^CD16^−^) was calculated for each subject by division between proportions of CD56^dim^CD16^high^/lymphocytes and CD56^dim^CD16^−^/lymphocytes.

### Health follow-up study

After the medical examination in 2007 and 2008, a questionnaire survey by telephone every year during 5 years following the initial tests was performed to determine whether the elderly subjects were alive or had died.

### Statistical analysis

All data were analyzed using the Windows Statistic Package for SPSS (version 100, Chicago, IL). Correlations between variables (cell types of NK cells and physical fitness parameters) were tested using the Spearman’s rank correlation. Body weight contributes to muscle force. Investigators suggested that the muscle force output, when measured with a dynamometer, might better reflect a force-generation impairment when normalized against body weight (Bohannon 2007; Eriksrud and Bohannon 2003). Therefore, the normalization by body weight was performed in every physical fitness data. To analyze the association of bilateral maximum knee extensor strength/weight (kg) compared to NK cell maturation, logistic regression models were used. The dependent variables consisted of the proportions of CD56^dim^CD16^high^ cells, CD56^dim^CD16^−^ cells, and CD56^high^CD16^−^ cells among the total CD56^+^CD16^+^ cells or lymphocytes. The BMI, smoking habits, drinking habits and medical history of each disease (cancer, diabetic mellitus, hypertension, cardiovascular diseases) and the bilateral maximum knee extensor strength/weight were used as independent variables for the analysis. A *P* value of 0.05 or less was considered statistically significant.

## Results

### Systemic parameters

Body height, weight, and BMI were 158.0 ± 3.4 and 147.9 ± 5.5 cm, 59.7 ± 17.1 and 48.9 ± 7.3 kg, and 23.8 ± 5.3 and 22.4 ± 3.0 kg/m^2^ in males and females, respectively. The data are at normal levels for healthy, elderly Japanese people. No detected information about drug use or diet was available, which may have biased the results through residual confounding. It seems unlikely, however, that these factors would account for all of the associations observed for knee strength, CD56^+^CD16^+^ cells and mortality.

### Correlation comparison for cell expression of CD56 and CD16 on lymphocytes and physical fitness

We correlated the relationship between the four types of cell subpopulations in Fig. 1 and physical fitness. In summary, all correlation data and statistical analyses showed a larger proportion of CD56^dim^CD16^high^/CD56^+^CD16^+^ cells having a significant positive correlation to the maximum right knee extensor strength/body weight (kg), the maximum bilateral knee extensor strength (kg) and the strength (kg)/body weight (r = 0.165, *P* = 0.027, 0.420 and 0.425, *P* < 0.0001) (Table 1). However, the proportion did not correlate with any other parameters. The proportion of CD56^dim^CD16^high^/lymphocytes and CD56^+^CD16^+^/lymphocytes correlated positively with the maximum hand grip strength (r = 0.179 and 0.167; *P* = 0.004 and 0.008, respectively) but other parameters did not. The proportion of CD56^dim^CD16^−^/lymphocyte population did not show significant correlations with any parameters. However, the ratio of CD56^dim^CD16^high^/CD56^dim^CD16^−^ cells correlated positively with the maximum bilateral knee extensor strength (kg) and strength (kg)/body weight (r = 0.317 and 0.323; *P* < 0.0001 and 0.0001, respectively) but did not correlate with the other parameters. The proportion of CD56^bright^CD16^−^/lymphocytes correlated negatively with the maximum bilateral knee extensor strength (kg) and strength (kg)/body weight (kg) (r = −0.290, *P* < 0.0001 and r = −0.256, *P* = 0.0004). The data normalized by body weight in physical fitness parameters excepting for maximum knee extensor strength did not correlate with various parameters of CD56^+^CD16^+/−^ cells. Other parameters for physical fitness, BMI, smoking habits, diabetes mellitus, hypertension, and cardiovascular disease did not show significant relationships with the various cell parameters. To analyze the association of maximum bilateral knee extensor strength/body weight with the proportion of CD56^dim^CD16^high^/CD56^+^CD16^+^ cells after adjustment with other confounding factors, logistic regression analyses were performed. Two logistic regression models showed maximum bilateral knee extensor strength/body weight to be independently associated with the proportion of CD56^dim^CD16^high^/CD56^+^CD16^+^ cells in both males and females (Table 2). The coefficient of variation for the unilateral knee extension strength on the right and left legs were respectively, 35.9 and 37.2 %, in the total elderly population. There was no significant difference between right and left leg strength. The proportion of CD56^dim^CD16^high^/CD56^+^CD16^+^ cells is useful as an indicator for the level of bilateral lower-muscle strength in all of the males and females.

**Table 1:** Correlation between physical fitness and natural killer cells types

Physical fitness parameters	CD56^+^CD16^+^/lymphocytes	CD56^dim^CD16^high^/lymphocytes	CD56^dim^CD16^high^/CD56^+^CD16^+^	CD56^dim^CD16^−^/lymphocytes	CD56^dim^CD16^high^/CD56^dim^CD16^−^	CD56^bright^CD16^−^/lymphocyte
Maximum hand grip (kg) (N = 247)	0.179**	0.167**	0.097	−0.105	0.088	−0.087
	*P* = 0.004	*P* = 0.008	*P* = 0.130	*P* = 0.097	*P* = 0.169	*P* = 0.171
One-leg standing time with eyes open (sec.) (212)	−0.042	−0.079	−0.079	0.008	−0.050	0.131
	*P* = 0.540	*P* = 0.251	*P* = 0.252	*P* = 0.904	*P* = 0.469	*P* = 0.056
Maximum stepping rate for 10 s (210)	0.100	0.043	0.040	0.316	0.027	0.084
	*P* = 0.224	*P* = 0.537	*P* = 0.566	*P* = 0.060	*P* = 0.695	*P* = 0.224
Maximum knee extensor strength (kg) right (184)	−0.001	0.045	0.136	0.060	0.080	−0.003
	*P* = 0.991	*P* = 0.547	*P* = 0.065	*P* = 0.420	*P* = 0.285	*P* = 0.659
Maximum knee extensor Strength/weight (kg) right (180)	−0.079	0.010	0.165*	−0.045	−0.033	0.001
	*P* = 0.291	*P* = 0.893	*P* = 0.027	*P* = 0.551	*P* = 0.662	*P* = 0.941
Maximum knee extensor strength (kg) left (184)	0.043	0.065	0.112	0.022	0.096	−0.058
	*P* = 0.565	*P* = 0.579	*P* = 0.131	*P* = 0.764	*P* = 0.195	*P* = 0.433
Maximum knee extensor Strength/weight (kg) left (180)	−0.026	0.030	0.128	−0.010	0.097	−0.045
	*P* = 0.726	*P* = 0.687	*P* = 0.086	*P* = 0.896	*P* = 0.197	*P* = 0.551
Maximum knee extensor Strength (kg) bilateral (193)	−0.050	0.150*	0.420**	−0.006	0.317**	−0.290**
	*P* = 0.490	*P* = 0.038	*P* < 0.0001	*P* = 0.933	*P* < 0.0001	*P* < 0.0001
Maximum knee extensor Strength/weight (kg) bilateral (188)	−0.105	0.108	0.425**	−0.026	0.323**	−0.256**
	*P* = 0.159	*P* = 0.149	*P* < 0.0001	*P* = 0.711	*P* < 0.0001	*P* = 0.0004
10-m maximum walking speed (203)	0.040	0.114	−0.034	0.021	0.028	−0.066
	*P* = 0.570	*P* = 0.106	*P* = 0.628	*P* = 0.814	*P* = 0.690	*P* = 0.349

*P value < 0.05

** *P* value < 0.01

**Table 2:** Association of bilateral maximal knee extensor strength weight (kg) with CD56^dim^CD16^high^/CD56^+^CD16^+^

Male	Female
6.55 (2.55–16.75)*	6.55 (2.57–21.78)*

Odds ratio (95 % confident intervals)

* P value <0.001

### Correlation comparison of expression of CD56 and CD16 on lymphocytes and physical fitness to mortality

Physical fitness has positive effects on the health of the elderly. It is therefore interesting to identify the relationship between physical fitness and NK cell parameters related to mortality. Elderly subjects were phoned yearly for 5 years to determine if they were alive following the medical and dental examinations in 2007 and 2008. Twenty seven elderly people (4 female and 23 male) died during the 5 years after the primary medical examination. The causes of death were 55.6 % (15/27) cancer (lung, gall bladder, stomach, colon, pancreas and prostatic), 0.7 % (2/27) pneumonia, 0.7 % (2/27) renal insufficiency, 0.4 % (1/27) malignant sarcoma, 0.4 % (1/27) cerebral hemorrhage, 0.4 % (1/27) heart failure, 0.4 % (1/27) emphysema, 0.4 % (1/27) acute coronary syndrome and 0.4 % (1/27) ureteral tumor. To determine if there was an association of death with NK cell surface marker or physical fitness, the proportion of the various types of cells and levels of physical fitness were compared between dead and alive groups.

**Fig. 2:**
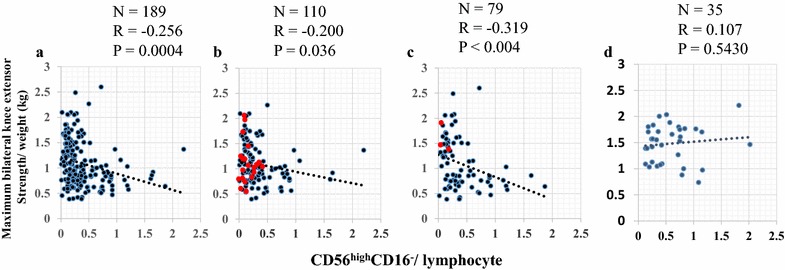
Scatter plot and correlation in relationships between maximum bilateral knee extensor strength/weight and CD56^bright^CD16^−^/lymphocytes. Correlation between maximum bilateral knee extensor strength/weight and CD56^bright^CD16^−^/lymphocytes was analyzed in total (**a**), female (**b**) and male (**c**) elderly, and young people (**d**). *Red color* *plot* indicates individual dead subjects during the 5 years following the medical examination. *N* numbers of subjects, *R* correlation rate. *P* significant correlation *P* value

The proportion of CD56^bright^CD16^−^/lymphocytes (0.177 ± 0.121 %) was significantly lower in the dead elderly compared to the living elderly (0.393 ± 0.345 %) but there were no significant differences in other cell types between the dead and living elderly. Therefore, it was considered that proportion of CD56^bright^CD16^−^/lymphocytes is a predictor for the risk of death. The correlation between CD56^bright^CD16^−^/lymphocytes and maximum bilateral knee extensor strength/body weight was investigated in the total female and male population. The proportion of CD56^brght^CD16^−^/lymphocytes had a significant negative correlation to the maximum bilateral knee extensor strength (kg)/body weight in all of the elderly (r = −0.256, *P* = 0.0004) (Fig. 2). The negative correlation was higher in females (r = −0.319, *P* = 0.004) and lower in males (r = −0.200, *P* = 0.036) (Fig. 2). The proportion of CD56^bright^CD16^−^/lymphocytes was associated inversely with the maximum bilateral knee extensor strength (kg)/body weight and was stronger in males than females. Red points were individually plotted as a dead elderly male or female (Fig. 2b, c). These red points were located less than 0.5 in the proportion of CD56^bright^CD16^−^/lymphocytes but there were no tendencies with the maximum bilateral knee extensor strength (kg)/body weight in these red points in male (Fig. 2b). In contrast, the red points were few (only 3), located less than 0.5 in the proportion of CD56^bright^CD16^−^/lymphocytes and more than 1.2 in the maximum bilateral knee extensor strength (kg)/body weight in females (Fig. 2c). Both averages of the proportion of CD56^bright^CD16^−^/lymphocytes and the maximum bilateral knee extensor strength (kg)/body weight were higher in young people than those in the elderly overall (Fig. 2a, d). There were no significant correlation (r = 0.107, *P* = 0.5430) between the proportion of CD56^bright^CD16^−^/lymphocytes and the maximum bilateral knee extensor strength (kg)/body weight in young people (Fig. 2d). Negative correlation between CD56^bright^CD16^−^/lymphocytes and the extensor strength was not observed in the young people who the proportion of CD56^bright^CD16^−^/lymphocytes (0.59 ± 0.43 %) was significantly higher in young people than those (0.37 ± 0.43 %) in elderly (*P* = 0.003).

## Discussion

Generally, people with mobility impairments tend to have a high morbidity and mortality rate that is caused by the deterioration of lower and upper skeletal muscle strength from reduced walking, eating and using the toilet (Yoshida et al. 2010). However, little is known about the interaction between the systemic increased mortality and physical fitness, especially regarding NK cells associated with protection against infection and tumor cell control in the very elderly.

Here, we found that CD56^dim^CD16^high^ cells included the mature NK and NKT cells were significant positive cellular immune indicators when compared to knee extensor and hand grip strength in very elderly subjects. The proportion of CD56^dim^CD16^high^ in CD56^+^CD16^+^ cells and the ratio of CD56^dim^CD16^high^ and CD56^dim^CD16^−^ cells correlated positively with the maximum bilateral knee extensor strength and maximum bilateral knee extensor strength/body weight. Logistic regression analysis showed the maximum bilateral knee extension strength/body weight was independently associated with the percentage of CD56^dim^CD16^high^ cells within the total CD56^+^CD16^+^ cells in males and females (Table 2). Various expression levels of CD16 were found on CD56 cells in the elderly. Reports show during maturation of the CD56^bright^ (immature cells) into CD3^−^CD56^dim^CD16^+^ NK cells (mature cells), the expression of NKp46, NKG2D, Nkp30, and NKG2A decreases and CD16 expression is acquired (Moretta et al. 2002). Thus, the amount of CD16 expression on the cell surface correlates with the level of maturation of the NK cell. CD3^+^CD56^+^CD16^+^ NKT cells also have cytotoxic activity. The CD56^dim^CD16^high^ cells have higher expression levels of CD16, which may be useful indicators of the maturation of the NK and NKT subpopulation cells in the elderly.

There are reports concerning the relationship between NK cell activity and physical activity. Kusaka et al. report that higher NK activity was found in individuals reporting beneficial life style practices who exercise once or more a week as a leisure-time activity (Kusaka et al. 1992). Further, moderate aerobic exercise training significantly increased NK cell activity in the elderly (Yan et al. 2001). This suggests that habitual moderate daily physical activity is beneficial for maintaining NK cell activity in older people who live independently (Senchina and Kohut 2007). Other reports show that daily physical activity is associated with muscle strength in very old people (Yoshida et al. 2010). This suggests that daily physical activity may be a factor contributing to the maintenance and/or increase of muscle strength in the very elderly. These studies partially support the association of NK cells with lower- and upper-limbs muscle strength observed in our present study, although CD16 expression-related NK cell activity was not associated with all muscle strength parameters. In particular, lower-muscle bilateral leg strengths are required for the relationship with the NK and NKT cell populations, but this association is not seen in other parameters such as one-leg standing time with eyes open, maximum stepping rate for 10 s and 10-m maximum walking speed test, etc. The bilateral knee extension strength is necessary to standing, which is one of physical activities in batteries of physical functioning (Eriksrud and Bohannon 2003). The combination and balance of right and left knee strength may be appropriate for physical activity resulting in the induction of differentiated NK and NKT cells.

In the correlation, NK parameters of immunity and muscle strength, the influence of immunity on the muscle strength is not considered because there are no significant data or reports on this thesis research. In contrast, the influence of the muscle strength on immunity is considered because that is supported by many reports. Therefore, the level of bilateral lower-muscle strength may be one factor that affects the proportion of NK and NKT cell such as CD56^dim^CD16^−^ cells and CD56^dim^CD16^high^ cells. This suggests that elderly people having reduced physical function, such as the bilateral knee extensor strength in the legs, tend to be susceptible to immunity-related diseases because they have less-mature NK and NKT cells (Renna et al. 2012; Small et al. 2008).

Cytolytic NK cells may control infected and tumor cells from infiltrating secondary lymphoid organs (Ferlazzo et al. 2004). The NK cells in secondary lymphoid organs can play a major role at enhancing patient resistance against malignancies (Ferlazzo and Münz 2004). Remagnan et al. suggest that CD56^bright^CD16^−^ and CD56^dim^CD16^+^KIR^;/−^ NK cells correspond to sequential steps of differentiation and support the hypothesis that secondary lymphoid organs may be sites for final NK cell maturation (Romagnani et al. 2007). Proportion of CD56^bright^CD16^−^ cells within lymphocytes associated negatively with the incidence of death. However, these cells lack perforin and killer cell Ig-like receptors (KIRs) and exist mainly in lymph nodes (Ferlazzo et al. 2004). In contrast, proportion of CD56^dim^CD16^high^ cells within lymphocytes did not significantly correlate with the incidence of death. CD56^dim^CD16^+^ cells express perforin, the natural cytotoxity receptor (NCRs) Nkp30 and Nkp46, as well as in-part KIRs (Ferlazzo et al. 2004; Jacobs et al. 2001; Romagnani et al. 2007). More than 95 % of the peripheral blood and 85 % of the spleen NK cells are CD56^dim^CD16^+^ cells (Ferlazzo et al. 2004). Exercise-induced increase in NK cells cytotoxicity is largely due to an increase in the absolute numbers and percentage of circulating blood NK cells (Walsh et al. 2011). IL-2 is required to induce natural cytotoxicity in these abundant NK cells. Cytokine-induced activation by IL-2 should be sufficient to induce natural cytotoxicity in these abundant NK cells harbored in solid tissues (Ferlazzo et al. 2004; Romagnani et al. 2007). On IL-2 secretion by T cells, NK cells up-regulate activating and inhibitory receptors as well as perforin and acquire the ability to kill NK-sensitive targets. However, subsequent NK cell activation by IL-2 stimulation was significantly impaired (Millard et al. 2013). Therefore, NK cells mobilized into the circulation in response to exercise have altered sensitivity to IL-2 stimulation (Millard et al. 2013). The circulating CD56^dim^CD16^+^ cells may also be altered and impaired in the subsequent activation by IL-2, and lose killing virus-infected and cancer cells. In contrast, CD56^bright^CD16^−^ cells can convert in CD56^dim^CD16^+^ cells able to kill virus-infected and cancer cells (Ferlazzo et al. 2004). Circulating CD56^bright^CD16^−^ cells may provide fresh cytotoxic cells into the tissues infected by viruses or tumors. Several reports demonstrate that antiviral treatment with interferon-α (IFN-α) increased the proportions of the CD56^bright^ subset, while those of the CD56^dim^ subset declined slightly (Fathy et al. 2010; Lee et al. 2010). They concluded that this may have advantages for patients because an increased secretion of IFNγ by the CD56^bright^ subset has an antiviral effect. Yamagiwa et al. suggests that a significant imbalance between CD56^bright^ and CD56^dim^ NK cell subsets in the liver may contribute to the progression of recurrent chronic hepatitis C after liver transplantation (Yamagiwa et al. 2014). Therefore, the differentiation from CD56^bright^CD16^−^ cells to CD56^dim^CD16^high^ cells may be responsible for the infected and tumor cells infiltrating secondary lymphoid organs and may be a key event to prevent spread of the infected and tumor cells in the very elderly. Taken together, the proportion of CD56^bright^C16^−^ cells/lymphocyte was found to be an important indicator of the incidence of death rather than CD56^dim^CD16^high^/CD56^+^CD16^+^ cells associated with the bilateral knee extensor strength in the legs in the very elderly.

Thus, bilateral knee extensor strength can predict differentiated NK and NKT cells to active cells forms. The susceptibility of the knee strength to the differentiation is higher in the elderly than that in young people. However, the activation by the knee strength did not associate with the mortality risk. The knee strength tends to decrease in CD56^bright^CD16^−^ cells/lymphocytes relating with incidence of death. Therefore, undifferentiated NK and NKT cells such as CD56^bright^CD16^−^ cells may be important to correspond to death by cancer, infection and inflammation, or be a usual indicator for mortality. Elderly should avoid consumption of undifferentiated NK and NKT cells by excessive exercise because its number is limited in elderly.

## Conclusion

The level of bilateral lower-muscle strength may be a factor that affects the proportion of NK and NKT cell such as CD56^dim^CD16^−^ and CD56^dim^CD16^high^ cells. The proportion of CD56^bright^C16^−^ cells within lymphocyte in peripheral blood is a predictor of incidence of death and correlated negatively with bilateral lower-muscle strength in the very elderly. Measurements of physical fitness, the proportion of CD56^dim^CD16^high^ cells in CD56^+^CD16^+^ cells, the ratio of CD56^dim^CD56^high^ and CD56^dim^CD16^−^ cells, and the proportion of CD56^bright^C16^−^ cells in lymphocytes are important indicators to check elderly health.
